# Complicated Abdominal Disease

**Published:** 1834

**Authors:** John Andrews

**Affiliations:** Steubenville, O.


### Art. III.—Complicated Abdominal Disease.

*An account of the last illness, death, and post mortem appearances, of the late Dr. Anderson Judkins, of Steubenville, Ohio.* By John Andrews, M. D.

The following case may present nothing new, either in its pathology or therapeutics. but as the subject of it was a most worthy member of the profession, and had many friends who are readers of the Western Journal, and reside in various parts of our State, a report of his case may not be inadmissible. Very few physicians in the West, were more anxious for the improvement and elevation of our science, and none surpassed him in active and expanded benevolence. Before the repeal of the medical law he was president of the 16th District Society, and one of the delegates to the General Medical Society. With these claims on our consideration, I venture to offer his case for publication.

Dr. Judkins, æt. 39 years, originally with a good constitution, and always remarkably temperate in his habits, had been engaged for the last 15 years in a laborious town and country practice, subjecting him to exposure and fatigue both of body and mind, but had generally enjoyed good health up to the time of the fatal attack, with the exception of habitual constipation of bowels and occasional attacks of colic; which were commonly slight and passed off, either by the unassisted efforts of nature, or by catharsis artificially induced. When subjected, in an unusual degree, to loss of sleep and anxiety of mind, he was sometimes liable to a febrile paroxysm, in which the brain was very apt to be disturbed in its functions, causing delirium; but a single general bleeding, cathar is, a foot bath, and perfect rest, invariably restored him to his wonted health, which might not be again interrupted for a period of from three to twelve months. His devotion to his profession, and his unceasing concern for his patients, caused him always to forget himself in his attention to others. Of late, his skin, naturally of a clear and sanguineous blush, had become slightly tinged with sallowness, and through the past year he had occasionally expressed an apprehension, "that all was not right in his liver," but no remedies were employed for its relief. In August last he experienced a sudden attack, attended with excruciating pains in the abdomen, with cold skin, small pulse and a paroxysm of complete syncope, soon followed by copious alvine and biliary discharges; after which, about half pint of a bright red secretion, resembling arterial blood, but not coagulable, was discharged by the rectum, in small quantities at a time, unmixed with any other matters. No febrile reaction ensued, and he attended to his business as usual on the following day; and from that time, uninterruptedly, up to the 4th of December. On that day, after eating a hearty supper, consisting, in part, of hot biscuit and fresh pork, he was attacked, at 9 o'clock, P. M., with severe pains in the umbilical region; attended with vomiting, great anxiety, jactitation, cold extremities, small pulse and costive bowels. His feet and legs were immersed in hot water, he was bled from the arm 24 ounces, had hot fomentations to the abdomen, took 10 grs. of calomel and 2 of opium, followed by ol. terebinth ʒi, ol. ricini ʒi combined; then ether, and enemata of a saturated solution of common salt, in an infusion of senna. The pains continued unabated until 1 o'clock, A. M., of the 5th, when he became somewhat composed and dozed until morning. At 9 A. M. the stomach was quiet, the bowels imperfectly moved, the abdominal pains, with tenderness on pressure continued, but less severe; the pulse 90, regular, compressible, and tongue dry in the centre, with thirst. A vein was again opened in the arm—the blood running freely at first, but soon failing, so that only about a pint was obtained. Calomel in 10 gr. doses every four hours, with other purgatives, enemata, and fomentations, were prescribed, to be used through the day until free purgation should be effected. At 9 P. M. no action upon the bowels, continued abdominal pain and soreness, lies with his legs flexed so as to relax the abdominal muscles, pulse 90. soft and regular, skin dry, not hot, tongue dry and clean. He was again bled from the arm, and about a pint of blood obtained, flowing, as before, in a manner which indicated visceral congestion and imperfect capillary circulation, the blood, after coagulation, exhibiting no buffy coat. Bitter extracts, castor oil and enemata were directed, and a large blister over the abdomen proposed, but hops boiled in vinegar substituted, with cold gum water acidulated as drink.

Dec. 6th, 8 A. M. Bowels freely moved three times in the night, stools dark and scybalous, after which enjoyed some rest, but has abdominal pain attended with remissions; pulse 80, soft and regular, skin cool, tongue dry. A bath was prepared of the temperature of 110°, into which, at 10 o'clock, he was immersed and remained 40 minutes, during which he was quite free from pain. When removed from the bath, his pulse being full, soft, and 90 in frequency; a vein was opened in the arm, and near a quart of blood taken *pleno rivo.* His skin perspired freely for about an hour afterwards. At 1 o'clock P. M., had a severe chill, which shook his whole body, and lasted 30 minutes; it was followed by general restlessness for a short time, but no increased frequency of pulse, and went off in a copious perspiration. At 9 P. M. there was tenderness of the abdomen on pressure, but no paroxysmal pains; tongue dry and brown in the centre; pulse 80. Cups were applied over the abdomen, which, however, did not bleed freely, and gentle laxatives, gum water and fomentations continued through the night.

Dec. 7th, 8 A. M. An enema at 12 at night had procured alvine evacuations, dark but not copious; felt some pain in the abdomen, and was desirous of a warm bath, which was allowed; after which he had (by his own direction) a large camphorated Burgundy pitch plaster, spread on thick leather, applied over the entire abdomen; took castor oil, and used acidulated gum water as a drink through the day, and in the afternoon, on account of chilliness, cal. and dov. powder, with infusion of serpentaria. At 9 P. M. his skin was universally moist, pulse 80, soft and regular; tongue more moist, but still rather dry in the centre; abdomen *perfectly free from pain on pressure*—which continued to be the case, from this period through the whole course of the disease. From this time, the chills formed a prominent feature in the general aspect of the case; they were ushered in suddenly, without premonition; sometimes seeming to be excited by the slightest exposure of the body to the air; sometimes by cold acid drinks taken into the stomach, but much oftener, without any, even suspected, cause whatever; varying in frequency, from once, in from 4 to once in 8 hours; in degree, from a mere sense of coolness, to a severe ague; and generally followed by great restlessness, some increase in the tension, none in the fulness or frequency of the pulse, followed in from five to twenty minutes, by excessive perspiration of the head and body, not of the extremities. With the chills, other symptoms appeared, such as severe and continued pains in the loins, excessive thirst, irritability of the stomach, which rejected drinks (gum water containing either acid or alkali) as if by a spasmodic action of the viscus itself; the fluids being unmixed with either alvine or hepatic excretions. The skin and adnata now exhibited a deep yellow tinge; the urine very high coloured and muddy or turbid after standing; the mind greatly depressed, foreboding a fatal issue to the case. The symptoms were supposed to indicate either mechanical obstruction of the hepatic ducts, or chronic disease of the liver, and the former view was adopted, perhaps with as much dependence upon the phenomena and termination of the attack, herein referred to, as occurring in August last, as upon the symptoms now present. The occurrence of the first chill, just 40 hours after the attack, was regarded as indicating irritation, rather than the formation of matter. The views of Marshall Hall,* on abdominal irritation, were regarded as throwing some light upon this question. Accordingly, the symptoms were treated with infusion of bone-set, (*Eupat. perfol.*) given twice as an emetic, at the approach of the chill, the warm bath, general bleeding, mercurial and oleaginous purgatives and *a large blister over the liver and stomach*, all which, varied to suit circumstances as they arose, were perseveringly employed for three days, viz. until the 11th, without the least advantage. The jaundice was now strongly marked; the tongue dry; stomach free from irritability; pulse 75, soft and regular; skin dry but cool; the alvine evacuations dark and floculent; the chills occurring as before, but followed by greater restlessness and much less moisture of the skin, and occasionally subsultus tendinum. It was now determined to withhold every thing from the stomach, which might be supposed capable, in the least degree, of irritating the gastro-intestinal surface, and to allow only cold gum water with a small portion of sup. carb. potas. as drink, and small portions of ice and injections of cold water to allay thirst, to establish extensive counter-irritation upon the skin, to attempt to control the chills by anodyne enemata, (water of assafœtida and laudanum,) to administer stimulating injections into the rectum, with the double view of producing counter-irritation and alvine evacuations. These general measures, adapting them to the varied circumstances of the case, were rigidly pursued up to the 25th, including a period of 14 days. Meanwhile there appeared to be some grounds for hoping, that they would prove successful—the alvine evacuations, procured by enemata, presented a natural appearance, being moulded and of a dark color. On two occasions, large flakes of false membrane were discharged with healthy excrement—indica-

* Vide Hall on "Intest. Irritation." p. 123 et seq. of his Treatise on "Loss of Blood."

ting effusion from the surface of the small intestines. The skin and eye lost their sallowness almost wholly; the serous discharge from the blistered surfaces was pellucid, and no longer stained linen; the chills, at all times more manageable, were entirely absent for a period of four days; the pulse seldom exceeded 70, never 80, in a minute, was always easily compressed and perfectly regular in its beats; the skin usually soft, but the tongue, although sometimes moist for a few hours, was generally dry, clean and red; and the urine high coloured and turbid after standing. Such was his general condition on the 25th. In the night following, without any apparent cause, he had a severe chill—and on the following morning his skin was *deeply jaundiced*. On the 26th, the chills recurred as in the early period of the disease; subsultus tendinum was frequently observed in the wrists; a careful examination of the abdomen detected obscure indications of the presence of the fluid in the peritoneal cavity. It was now determined to effect a ptyalism, and accordingly 2 grs. calomel, with ½ gr. squill was given every three hours, and the blisters, generally, were dressed with mercurial ointment, and frictions with it, used to the arms and legs, the bowels being still preserved regular by enemata, and the chills counteracted, or moderated, by anodynes, used in the same way. These means proved wholly ineffectual: the constitution of the patient seemed to be wearing down under continued irritation; he had occasional singultus, producing severe pain, flushes of redness in one or both cheeks, hectic pulse, and profuse sweats after a chill. Ablutions of dilated nitro-muriatic acid, and dressing part of the blisters with sulph. quinine were added to the other means, when the profuse sweats entirely ceased.

On the 5th of January, an attempt was made to increase the quantity of calomel to 5 grs. every three hours—but the stomach rejected it soon; a green fluid, in large quantity, was thrown off from the same organ; two or three copious, liquid, black stools were discharged by the rectum. The singultus was more frequent. On the morning of the 6th, the breath was very offensive, sordes accumulated upon the teeth, flakes of viscid mucous were discharged from the mouth, the tongue perfectly crisped. On the 7th the mental faculties were disturbed; he exhibited loss of sensibility, by expressing a conviction of his ability to rise and set up, and also a desire for food with great hunger. From this time he sunk rapidly, and quietly breathed his last at 4 A. M. of the 8th.

Permission being granted, the body was inspected, and presented the following morbid appearances.

1. General emaciation, with prominence of abdomen.

2. Sallowness of the skin, the face, particularly, having a deep saffron tinge. The adipose and serous tissues, exposed by opening the cavity of the abdomen, were similarly discolored.

3. The peritoneal cavity contained a quantity of serum, slightly approaching the character of a sero-purulent fluid, with a portion of black coloring matter in it, estimated at three pints.

4. The greater omentum adhered by a strong connexion, two inches long, to the posterior or free surface of the peritoneum, lining the abdominal parieties, above the symphisis pubis.

5. The contiguous surfaces of the small intestines were agglutinated, in many places, but the false adhesions about the duodenum were particularly firm and extensive.

6. The duodenum, externally, was of a dirty white, or ash color, its coats easily perforated with the finger, containing a dark fluid like coffee grounds, in small quantity; and its mucous coat perfectly soft and easily rubbed off. The light was insufficient to note accurately minute objects.

7. The jejunum and ileum, externally, exhibited a bright red blush, as of parts fully injected with red blood. They contained a dark, jelly-like fluid, in small quantity, similar to what had been evacuated the two last days. The mucous coat was red in patches, but firm, as was the muscular also.

8. The colon was not connected by any false adhesions; its coats were of a healthy appearance, and contained flatus and healthy mucus only. Upon the posterior surface of its arch, there was an oval shaped deposit of lymph, of a black color, exterior to the peritoneal coat, about two inches long, a line thick, and its margins perfectly smooth and defined; with a few dark flakes, resembling coagulated blood, hanging from its free surface. The effusion was very firm, and the intestine otherwise healthy.

9. The stomach was bound down, by means of the greater omentum, to the intestines—was moderately distended with flatus, and its inner surface smeared over with a tough, viscid, light green coat of mucus, precisely similar to what was vomited. The mucous coat was of an ash color, was quite soft and easily scraped off with the finger nail, but was free from any patches or points of inflammation.

10. The head of the pancreas was firmly and closely united to the posterior surface of the duodenum—it was enlarged and very hard—*it seemed to compress the ductus communis choledichus*, which, in fact, was not traced beyond this point, but I am not satisfied that it was impervious.

11. The gall bladder was moderately distended, and adhered very firmly to the duodenum. It contained a straw colored fluid, with a black pigment diffused throughout, but entirely distinct from it—the inner coat was highly vascular, the vessels being injected with red blood.

12. The convex surface of the liver had a few thread-like adhesions to the corresponding surface of the diaphragm. The viscus was not enlarged, was of a dirty ash color, and upon the convex surface of the right lobe, there were a number of irregular, granulated eminences, an inch in diameter projecting about one line from the smooth or even surface. Three or four of these were clustered together, in the point formed by the inferior edge of the lobe, and the great fissure, and the slightest touch of the scalpel opened their cavities, which were filled with a thick, ropy, curd-like fluid, of a yellow tinge, each cavity containing about a tea spoonful—but the cavities of those clustered at the margin, communicated with each other, and formed a collection of about ℥j. The left lobe was healthy in its appearance, the peritoneal coat of the concave surface, and its reflection, forming the lesser omentum, in an inflamed state, forming false adhesions with the adjacent parts.

13. The spleen and kidneys perfectly healthy, and free from false adhesions.

14. The bladder empty; the cavity of the pelvis filled with serous effusion.

*Remarks.*—The inferences deducible from these facts would seem to be:—

1. That *chronic enlargement of the head of the pancreas* had existed prior to the present attack—occasioning obstruction to the passage of bile through the common duct, into the duodenum—which accounts for the habitual constipation of the bowels, and sallowness of the skin.

2. That the remora of fluids thus produced in the liver, had gradually developed tubercular disorganization of this important viscus—a disease which Dr. Abercrombie, in his invaluable work "On the stomach," says, "produces marked symptoms, only when the tubercles are numerous, or accompanied by enlargement of the liver, or disease of its general structure; but that when the structure is otherwise healthy, *they may exist without any symptom calculated to produce a suspicion of their presence*," and it is quite probable that such was the character of the disease of the liver which existed in this case.

3. That extensive inflammation of the stomach, small intestines (duodenum particularly) and peritoneum, was the cause of death; but that, in all probability, the "*fons et origo mali*," was the enlarged head of the pancreas, exciting inflammation of the gall ducts and duodenum—and extending thence by continuous sympathy, to the other organs involved. Fœcal accumulations and irritating ingesta, may have served as the exciting cause of the present attack.

4. That the *tongue* was the safest and truest index of the nature of the disease—and that the pulse was, what Hippocrates describes it, "*res fallacissima*," in the present instance, confirming the important remark, "that the pulse is a very uncertain index of the condition of the parts in enteritis and peritonitis, being often slightly affected through their whole progress to fatal disorganization.

5. That, upon the foregoing premises, the disease was, at the time of the attack, incurable by any means known to the profession; for what could act upon the head of the pancreas?

In the progress of the disease, the question was often discussed in consultation, and it may not be improperly here repeated, "Whence proceeded, and to what shall we attribute the chills?" unquestionably the most distressing circumstance to the patient attending the disease. Two explanations were given—1. That they depended upon, and were indicative of, the formation of abscesses in the liver. 2. That they proceeded from irritation of the abdominal viscera, and that upon the principle "ubi irritatio, ibi affluxus," blood was withdrawn from the surface and concentrated upon the viscera, producing chills in the same manner as a cold bath, or intermittent fever.

Those who adopted the former view, will probably regard the matter found in the liver, as confirmatory of its correctness. Those who entertained the latter would probably oppose something like the following objections. 1. The softening of tubera is a very distinct disease from the formation of abscess in the liver—and that it is more consistent with facts to say, that chills do not, than that they do, attend the former disease. 2. That the first chill, occurring 40 hours after the attack, was too early in the disease for the formation of matter. 3. That the matter found, was the result of chronic action, and was in existence *at the time of the attack*. 4. That the extent of the congestive and inflammatory action, was quite sufficient to produce such chills—which, when dependent upon this cause, might occur frequently, from the first onset, to the final termination of the disease.

P. S. Drs. Dickson and Scott, with myself, were his attending physicians. And Drs. J. C. Campbell of Wellsburg, M'Bean of Cadiz, Bates of Smithfield, and Hamilton of Mount Pleasant, gave us the benefit of their advice in consultation.

*Steubenville*, O., *Jan.* 17, 1835.

